# Parallel fiber to Purkinje cell synaptic impairment in a mouse model of spinocerebellar ataxia type 27

**DOI:** 10.3389/fncel.2015.00205

**Published:** 2015-06-04

**Authors:** Filippo Tempia, Eriola Hoxha, Giulia Negro, Musaad A. Alshammari, Tahani K. Alshammari, Neli Panova-Elektronova, Fernanda Laezza

**Affiliations:** ^1^Department of Pharmacology and Toxicology, University of Texas Medical BranchGalveston, TX, USA; ^2^Department of Neuroscience, University of TorinoTorino, Italy; ^3^Neuroscience Institute Cavalieri OttolenghiTorino, Italy; ^4^National Institute of Neuroscience—TorinoItaly; ^5^Pharmacology and Toxicology Graduate Program, University of Texas Medical BranchGalveston, Texas, USA; ^6^King Saud University Graduate Studies Abroad ProgramRiyadh, Saudi Arabia; ^7^Mitchell Center for Neurodegenerative Diseases, University of Texas Medical BranchGalveston, TX, USA; ^8^Center for Addiction Research, University of Texas Medical BranchGalveston, TX, USA; ^9^Center for Biomedical Engineering, University of Texas Medical BranchGalveston, TX, USA

**Keywords:** ataxia, synaptic transmission, glutamatergic synapse, parallel fiber, purkinje cell, cerebellum

## Abstract

Genetically inherited mutations in the fibroblast growth factor 14 (FGF14) gene lead to spinocerebellar ataxia type 27 (SCA27), an autosomal dominant disorder characterized by heterogeneous motor and cognitive impairments. Consistently, genetic deletion of *Fgf14* in *Fgf14*^−/−^ mice recapitulates salient features of the SCA27 human disease. *In vitro* molecular studies in cultured neurons indicate that the *FGF14*^F145S^ SCA27 allele acts as a dominant negative mutant suppressing the FGF14 wild type function and resulting in inhibition of voltage-gated Na^+^ and Ca^2+^ channels. To gain insights in the cerebellar deficits in the animal model of the human disease, we applied whole-cell voltage-clamp in the acute cerebellar slice preparation to examine the properties of parallel fibers (PF) to Purkinje neuron synapses in *Fgf14*^−/−^ mice and wild type littermates. We found that the AMPA receptor-mediated excitatory postsynaptic currents evoked by PF stimulation (PF-EPSCs) were significantly reduced in *Fgf14*^−/−^ animals, while short-term plasticity, measured as paired-pulse facilitation (PPF), was enhanced. Measuring Sr^2+^-induced release of quanta from stimulated synapses, we found that the size of the PF-EPSCs was unchanged, ruling out a postsynaptic deficit. This phenotype was corroborated by decreased expression of VGLUT1, a specific presynaptic marker at PF-Purkinje neuron synapses. We next examined the mGluR1 receptor-induced response (mGluR1-EPSC) that under normal conditions requires a gradual build-up of glutamate concentration in the synaptic cleft, and found no changes in these responses in *Fgf14*^−/−^ mice. These results provide evidence of a critical role of FGF14 in maintaining presynaptic function at PF-Purkinje neuron synapses highlighting critical target mechanisms to recapitulate the complexity of the SCA27 disease.

## Introduction

FGF14 is a member of the intracellular fibroblast growth factor family comprising four gene products (FGF11–FGF14) and their respective splice isoforms (Itoh and Ornitz, 2008). Despite sharing sequence and structural homology with the canonical, secreted FGFs, none of the iFGFs have been reported to act through tyrosine-kinase FGF receptors (Schoorlemmer and Goldfarb, 2001), suggesting functional divergence between the two groups (Itoh and Ornitz, 2008). iFGFs have recently gained emerging interest in the context of normal brain function and brain disorders (Hsu et al., 2014).

Milestone studies in this field began with the identification of the naturally occurring *FGF14*^F145S^ mutation as the hereditary cause of spinocerebellar ataxia 27 (SCA27) in a large three-generation Dutch family (van Swieten et al., 2003; Brusse et al., 2006). SCA27 is a debilitating childhood-onset condition characterized by postural tremor, slowly progressive ataxia, and cognitive deficits. Further genetic analyses corroborating the initial findings revealed frameshift and amino acid exchange mutations, deletions, (Dalski et al., 2005; Coebergh et al., 2014) and chromosome translocations (Misceo et al., 2009). In addition, it revealed single nucleotide polymorphisms of *FGF14* (Dalski et al., 2005; Chen et al., 2012) in SCA27 patients unrelated to the initial family, extending the role of *FGF14* to a broader group of human subjects. Apart from SCA27, evidence of FGF14 chromosomal translocations has also been found in a patient afflicted with paroxysmal non-kinesigenic dyskinesia (PNKD; Shimojima et al., 2012) expanding the repertoire of human disorders linked to *FGF14*.

A flourishing body of studies in recombinant cell systems and in native tissue has demonstrated that the FGF14 wild type protein is an accessory subunit of the voltage-gated sodium (Nav) channels (Nav1.1–1.9; Lou et al., 2005; Laezza et al., 2007, 2009; Goetz et al., 2009; Shavkunov et al., 2012, 2013; Ali et al., 2014; Hsu et al., 2014, 2015). Through a high-affinity direct interaction with the intracellular C-tail of Nav channels, FGF14 acts as an isoform-specific modulator of Nav channels producing effects on peak current density, and voltage-dependence of activation and inactivation of Na^+^ currents (Lou et al., 2005; Laezza et al., 2007, 2009; Shavkunov et al., 2013). In neurons, FGF14 co-localizes with native Nav channels at the axonal initial segment (AIS), an interaction that is required for the reciprocal targeting of the two proteins (FGF14 and Nav channels) and is regulated by the activity of kinases (Lou et al., 2005; Laezza et al., 2007, 2009; Shavkunov et al., 2012, 2013; Xiao et al., 2013; Hsu et al., 2015).

The mechanism of action of the SCA27 mutation has been attributed to a dominant negative suppressive role of the *FGF14*^F145S^ mutated gene product over the FGF14 wild type protein. Support for this hypothesis comes from in-cell studies showing that the *FGF14*^F145S^ mutation decreases Nav currents and reduces neuronal excitability in hippocampal neurons by disrupting FGF14 and Nav channel localization at the AIS, phenotypes attributed to cellular sequestration and thereby suppression of activity of the FGF14 wild type protein induced by the SCA27 mutant (Laezza et al., 2007). Further support for this model comes from animal studies. Genetic deletion of *Fgf14* in *Fgf14*^−/−^ mice leads to severe ataxia, paroxysmal dystonia, and cognitive impairment (Wang et al., 2002; Wozniak et al., 2007) associated with deficits in synaptic plasticity in the hippocampus (Xiao et al., 2007) and neuronal excitability in the cerebellum (Goldfarb et al., 2007; Shakkottai et al., 2009). The remarkable similarity and complexity in phenotypes between SCA27 patients and *Fgf14*^−/−^ mice corroborates the dominant negative hypothesis of the *FGF14*^F145S^ mutation, but raises the question of whether suppressing Nav channel function (through loss of FGF14) is the solely cause of SCA27. Pioneering studies suggest that at least in the hippocampus other mechanisms, independent of Nav channel function including changes in the pre-synaptic release machinery might be concomitant causes of the disease (Xiao et al., 2007).

More recently *in vitro* studies have come to similar conclusions showing that overexpression of the FGF14^F145S^ mutant or silencing of *Fgf14*, results in loss of presynaptic Ca^2+^ currents and reduced glutamate release in cultured cerebellar neurons, a mechanism that has been ascribed to regulation of presynaptic voltage-gated Ca^2+^ (Cav) channels by the FGF14 wild type protein (Yan et al., 2013). Altogether, these *in vitro* studies in cultured cerebellar cells and in the intact hippocampus point out a major role of FGF14 in regulating synaptic transmission by mechanisms that are not directly related to Nav channels. However, it remains to be determined whether parallel fibers (PF) to Purkinje neuron synapses, the primary output of cerebellar circuit and the most vulnerable synaptic connection in ataxias, are affected by genetic deletion of *Fgf14* and exhibit any phenotype consistent with *in vitro* studies.

As a result of our study we found that PF to Purkinje neuron synapses in *Fgf14^−/−^* mice exhibit deficits in synaptic transmission and increase in pair-pulse facilitation associated with a decreased expression of the presynaptic marker VGLUT1, a specific marker of PF presynaptic terminals to Purkinje neurons. Overall, these data provide new knowledge on the FGF14 role in human disorders and highlights its multifaceted role for synaptic function in the brain.

### Methods

#### Animal Care

*Fgf14^−/−^* mice were maintained on an inbred C57/BL6J background (greater than ten generations of backcrossing to C57/BL6J) Animals were bred in the UTMB animal care facility by mating either heterozygous *Fgf14*^+/−^ males and females or homozygotes *Fgf14*^−/−^ males with *Fgf14*^+/−^ females. The University of Texas Medical Branch operates in compliance with the United States Department of Agriculture Animal Welfare Act, the Guide for the Care and Use of Laboratory Animals, and IACUC approved protocols. Another lot of animals was bred in the Neuroscience Institute Cavalieri Ottolenghi animal facility and the experiments were approved by the Bioethic Committee of the University of Torino (September 14, 2011) and by the Italian Ministry of Health (October 17, 2011). Mice were housed, *n* ≤ 5 per cage, with food and water *ad libitum*. All genotypes described were confirmed by PCR analysis conducted either in house or by Charles River Laboratories International, Inc.

#### Electrophysiological Recordings

Adult *Fgf14*^−/−^ and wild-type littermates (>2 month old) were anesthetized with isoflurane (Isoflurane USP, Webster Veterinary) and decapitated. Parasagittal cerebellar slices (200 μm) were sectioned using a vibratome. After recovery (1 h) at room temperature, slices were continuously perfused at 2–2.5 ml/min with an artificial cerebrospinal fluid (ACSF) containing (in mM): 125 NaCl, 2.5 KCl, 2 CaCl_2_, 1 MgCl_2_, 1.25 NaH_2_PO_4_, 26 NaHCO_3_, 10 or 20 glucose, bubbled with 95% O_2_/5% CO_2_ (pH 7.4). All recordings were performed at room temperature. Whole-cell voltage-clamp recordings were obtained from the Purkinje cells (PC) soma using borosilicate glass pipettes (2.0–3.5 MΩ). PCs were voltage clamped at −70 mV using an Axopatch 200 A amplifier (Molecular Devices), low pass filtered at 2 KHz and digitized at 10 KHz. The internal solution contained (in mM): 140 K-methanesulfonate, 10 HEPES, 4 MgATP, 0.4 Na_3_GTP, 0.5 EGTA, pH 7.3 (adjusted with KOH). To evoke excitatory postsynaptic currents (EPSCs) derived from the parallel fiber-Purkinje cell synapse (PF-EPSCs), square pulses (100 μs) were applied through a monopolar tungsten electrode (FHC, Bowdoin, ME, USA) placed in the molecular layer. Asynchronous EPSCs were recorded with an EPC9 amplifier (HEKA Elektronik, Lambrecht/Pfalz, Germany), filtered at 3 kHz and digitized at 10 kHz, with an internal solution containing: 130 CsCl, 4 MgCl_2_, 10 HEPES, 4 Na_2_ATP, 0.4 Na_3_GTP, 10 EGTA, 5 N-(2, 6-dimethylphenyl)acetamide-2-triethylammonium bromide (QX-314), pH 7.2 (adjusted with CsOH). Quantal analysis was performed on asynchronous EPSCs within a ~50–200 ms window following evoked synaptic events. All recordings were performed in the presence of the GABA_A_ antagonist gabazine (SR 95531, 20 μM) in the saline solution to rule out any contribution of fast IPSCs in response to GABAergic fibers activation. Slow-EPSCs mediated by the mGluR1 receptor were recorded in the presence of a cocktail of (2, 3-dihydroxy-6-nitro-7-sulfamoyl-benzo[f]quinoxaline-2, 3-dione) (NBQX 10 μM) and DL-2-Amino-5-phosphonovaleric acid (DL-AP5, 50 μM) to block ionotropic glutamate receptors. All drugs were purchased from Tocris and applied via the chamber perfusion line.

### Immunohistochemistry

*Fgf14*^−/−^ and *Fgf14*^+/+^ mice (2–4 months old) were deeply anesthetized with a ketamine/xylazine cocktail (100 mg/kg ketamine and 10 mg/kg xylazine, i.p.) and transcardially perfused with 4% formaldehyde freshly prepared from paraformaldehyde powder in phosphate buffer, pH 7.4. Brains were removed and post-fixed overnight in the same fixative at 4°C, followed by cryoprotection in 30% sucrose in 0.1 M phosphate buffer at 4°C. Brains were embedded in OCT, and sagittal cryostat sections of cerebellum (30 μm). Samples were washed with PBS, permeabilized with 1% Triton X-100, 0.5% Tween 20 in PBS, blocked for 30 min in PBS containing 5% normal donkey serum (NDS), and incubated overnight at 4°C with the following primary antibodies: rabbit anti-vescicular glutamate transporter 1 (VGLUT1, 1: 1,500, Synaptic System), rabbit anti-vesicle associated membrane protein 2 (VAMP2, 1:300, Vinci Biochem) and rabbit monoclonal anti-metabotropic glutamate receptor 1 (mGluR1, 1:800, Euroclone) dissolved in TBS containing 1, 5% NDS. Samples were then washed and incubated for 1 h with the following secondary antibodies at a 1:500 dilution: Alexa 488-conjugated donkey-anti-rabbit, and Alexa 555-conjugated donkey-anti-rabbit.

### Imaging Studies

Part of the confocal images were acquired with a Zeiss LSM-510 Meta confocal microscope using a multi-track acquisition with excitation lines at 488 nm for Alexa 488 and 543 nm for Alexa 568. Respective emission filters were band-pass 505–530 nm, band-pass 560–615 nm. Optical slices were 2 μm with a frame size of 512 × 512, pixel time of 6.40 μs, pixel size 0.28 × 0.28 μm and a 4-frame Kallman–averaging. A second set of images was acquired with a Nikon D-ECLIPSE C1Si confocal microscope using a multi-track acquisition with excitation lines at 488 nm for Alexa 488 and 543 nm for Alexa 555. Respective emission filters were band-pass 505–530 nm and band-pass 560–615 nm. Confocal Z-stacks for this set were collected at 2 μm or 0.5 μm steps with a frame size of 1024 × 1024, pixel time of 1.68 μs, pixel size 207.2 nm. All acquisition parameters, including photomultiplier gain and offset, were kept constant throughout each set of experiments. Acquired Z-stacks were sum-projected and pixel intensity values from the resulting stacked TIFF images were analyzed with ImageJ (NIH). Puncta analysis was performed using in-house macros developed for ImageJ with the following parameters: range size of 2–10 pixel units, threshold between 9% and 11%, fixed ROI for the molecular layer. For the mGluR1 analysis, fixed size ROIs were superimposed to Alexa 488 images corresponding to mGluR1 immunoreactivity and used to capture mean fluorescence values.

### Synaptic Membrane Preparation and Western Blot Analysis

Brain homogenates and synaptosomes were isolated from homogenized cerebellar tissue using a combination of sucrose gradient layering and ultracentrifugation as described in previous studies (Bjorklund et al., 2012). For Western blot analysis, protein samples from homogenate and synaptosomes were measured using Pierce™ BCA Protein Assay Kit (Thermo Fisher Scientific). Equal amount of proteins (30 mg/lane) were resuspended in 4× sample buffer containing 50 mM tris(2-carboxyethyl)phosphine (TCEP). Mixtures were heated for 10 min at 56°C and resolved on 4–15% polyacrylamide gels (BioRad, Hercules, CA). Proteins were electrophoretically transferred onto a PDVF membrane (Millipore, Bedford, MA) for 1.5–2 h at 4°C, 75 V. Membranes were blocked with 2% non-fat dry milk in TBS and 0.1% Tween-20 for 30 min and probed with the following primary antibodies: mouse anti-VGLUT1 (1:1,000, NeuroMabs), mouse anti-GluR2 (1:1,000, Millipore), mouse anti-mGluR1 (1:1,000, NeuroMab) dissolved in blocking solution. Membrane were probed first with anti-VGLUT1, then gently stripped for ~5 min with stripping buffer (Thermo Scientific, catalog number 46,430) and re-probed with anti-GluR2 and anti-mGluR1. Washed membranes were incubated with goat anti-mouse HRP antibody (1:5,000–10,000; Vector Laboratories, Burlingame, CA) and detected with ECL Advance Western Blotting Detection kit (GE Healthcare, Piscataway, NJ); protein bands were visualized using FluorChem® HD2 System and analyzed using the publically available ImageJ/Fiji (**fiji**.sc/) software. Rectangular areas were drawn and overlaid on each band and mean gray intensity values of high resolution. TIFF imaging files were used for quantification.

### Statistics

Data are presented as mean ± SEM. For data that passed the normality test, the statistical comparison was performed using unpaired two-tail Student’s *t*-test. Data for which the normality test failed were compared by the Mann–Whitney *u*-test. Data were tabulated and analyzed with Excel, Origin 8.6 (OriginLab Corporation, Northampton, MA USA). *P* values less than 0.05 were considered significant.

## Results

Previous studies have established that the *Fgf14^−/−^* mouse model recapitulates an array of motor and cognitive phenotypes that are found in humans carrying the *FGF14*^F145S^ mutation (Wang et al., 2002), the genetically inherited cause of SCA27 (van Swieten et al., 2003; Brusse et al., 2006). The primary mechanistic explanation of the disease has been attributed to decreased activity of Nav channels with consequent impairment of neuronal excitability (Goldfarb et al., 2007; Shakkottai et al., 2009) via suppression of the FGF14 wild type activity (Laezza et al., 2007). However, changes in presynaptic function, independent of Nav channels, might contribute to the disease phenotype (Yan et al., 2013).

**Figure 1:**
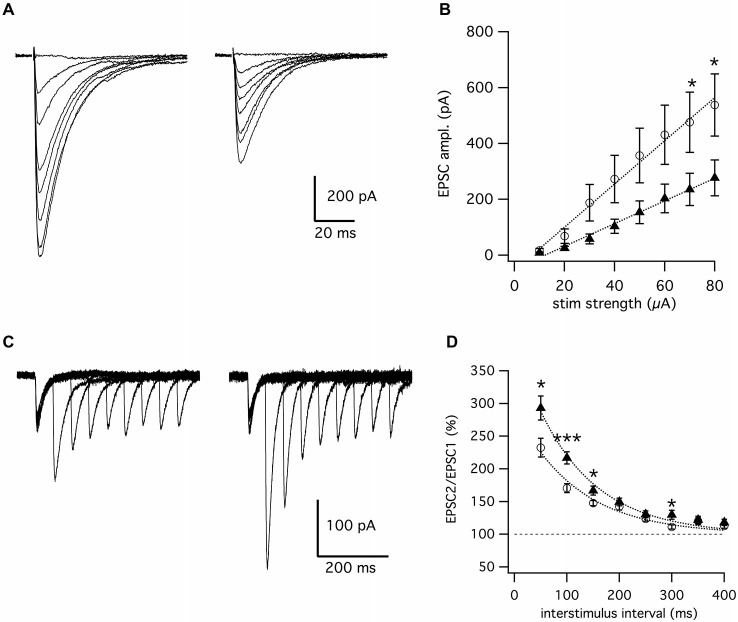
**Genetic deletion of *Fgf14* suppresses synaptic transmission at parallel fibers to Purkinje cell synapses. (A)** Representative superimposed traces of EPSCs evoked by parallel fiber stimulation with 10–80 μA from *Fgf14*^+/+^ (left) and *Fgf14*^−/−^ mice (right). **(B)** Input-output curve representing EPSC mean amplitude vs. parallel fibers stimulation with linear fitting curves. Note the reduction of EPSCs responses in *Fgf14*^−/−^ (*n* = 18, filled triangles) in comparison to *Fgf14*^+/+^ (*n* = 20, open circles). **(C)** Superimposed traces of EPSCs evoked by paired-pulse stimulation of parallel fibers with interpulse intervals from 50 to 400 ms in *Fgf14*^+/+^ (left) and *Fgf14*^−/−^ mice (right). **(D)** Time course of paired-pulse facilitation in *Fgf14*^+/+^ (*n* = 14, open circles) and *Fgf14*^−/−^ mice (*n* = 18, filled triangles) with exponential fitting curves. Note that PPF at short inter-stimulus intervals, PPF is significantly higher in *Fgf14*^−/−^ (filled triangles) in comparison to *Fgf14*^+/+^ mice (open circles); data are mean ± SEM; *: *P* < 0.05; ***: *P* < 0.001.

To this end, we applied whole-cell voltage-clamp in the acute cerebellar slice preparation to examine the properties of the PF to Purkinje neuron synapses in *Fgf14^−/−^* mice and age-matched wild type littermates. To ensure full manifestation of the early-onset SCA27 disease, animals selected for this study were older than 2 months, the age at which the knock-out animals exhibit visible ataxia, paroxysmal dyskinesia and other reported motor and cognitive abnormalities (Wang et al., 2002; Wozniak et al., 2007). The excitatory glutamatergic synaptic responses in the Purkinje neurons were induced by electrical stimulation of the PF by placing the stimulating electrode in the molecular layer. Extracellular synaptic stimulation evoked fast excitatory postsynaptic responses (EPSCs) mediated by activation of α-amino-3-hydroxy-5-methyl-4-isoxazolepropionic acid receptors (AMPA-R) in both animal groups (Figure 1A), albeit EPSCs were visibly smaller in amplitude in *Fgf14*^−/−^ mice. The reduction in amplitude was evident at virtually all extracellular stimulation strengths ranging from 10 to 80 μA, but the separation between the two groups was progressively higher as the stimulus intensity increased, reaching statistical significance at high stimulation intensities (60–80 μA). As illustrated in Figure 1B, the EPSCs input-output curve in *Fgf14*^−/−^ mice was significantly reduced compared to wild type mice (*Fgf14*^+/+^ *n* = 20; *Fgf14*^−/−^ *n* = 18 cells, *p* < 0.05, Mann-Whitney test), indicating that deletion of the* Fgf14* gene has a significant impact on basal synaptic transmission at PF to Purkinje neuron synapses.

To determine whether these changes were associated with functional deficits in presynaptic glutamate release and short-term synaptic plasticity, we measured synaptic responses evoked by paired-pulse stimulations of variable inter-pulse intervals. As shown in Figures 1C,D, while modest paired-pulse facilitation (PPF) was observed at wild type synapses, PPF was significantly higher in *Fgf14^−/−^* animals, especially for stimuli delivered at short inter-pulse intervals. At a 50 ms interval, PPF was 232 ± 14% (*n* = 14) in wild type and 293 ± 18% (*n* = 18) in *Fgf14*^−/−^ mice (*p* < 0.05, Mann-Whitney test); at 100 ms PPF was 170 ± 7% (*n* = 14) in wild type and 217 ± 9% (*n* = 18) in *Fgf14*^−/−^ mice (*p* < 0.001, Mann-Whitney test); and a 150 ms PPF was 148 ± 4% (*n* = 14) in wild type and 168 ± 7% (*n* = 18) in *Fgf14*^−/−^ mice (*p* < 0.05, Mann-Whitney test). Given that PPF is an accepted measure of presynaptic function attributed to residual Ca^2+^ content in the presynaptic terminal (Wu and Saggau, 1994; Debanne et al., 1996; Scullin et al., 2012; Bornschein et al., 2013), these results suggest that PF to Purkinje neuron *Fgf14*^−/−^ connections exhibit impaired basal synaptic transmission associated with disrupted presynaptic function.

**Figure 2:**
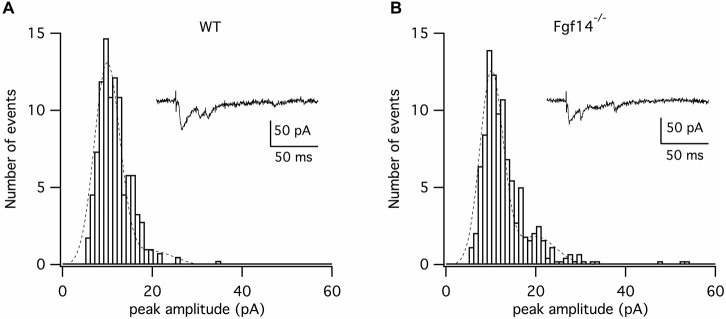
**Asynchronous quantal PF-EPSCs are not affected by genetic deletion of *Fgf14***. Cumulative plots of asynchronous EPSCs size following parallel fiber stimulation. Amplitude histograms were fitted (dashed lines) by a multipeak gaussian function to detect the size of unitary quantal events of *Fgf14*^+/+^ **(A)** and *Fgf14*^−/−^ **(B)** mice. Representative traces are shown in the insets.

To strengthen this hypothesis, we performed quantal analysis of miniature PF-EPSCs. Due to the mixed nature of spontaneous miniature EPSCs in PC, arising from both PF and climbing fiber synapses, the analysis was performed on miniature PF-EPSCs occurring during asynchronous release of quanta induced by stimulation of the PF synapse. To this purpose, minis were recorded in the presence of extracellular Sr^2+^ (3 mM), in lieu of Ca^2+^, to stimulate asynchronous neurotransmitter release. As shown in Figure 2, the distribution of the amplitudes of asynchronous PF-EPSCs was fitted by a multi-peak gaussian function, which detected a first peak at 9.9 pA in wild type (*n* = 4) and at 10.2 pA in *Fgf14*^−/−^ PCs (*n* = 5), corresponding to 0.142 nS and 0.145 nS of quantal conductance, respectively. These values are in accordance with previously reported data (Sims and Hartell, 2005; Valera et al., 2012). The lack in the difference between the quantal conductance in the two experimental groups indicates that the mechanism underlying the decreased PF-EPSCs amplitude in *Fgf14*^−/−^ mice is unlikely attributable to a reduction in postsynaptic sensitivity.

**Figure 3:**
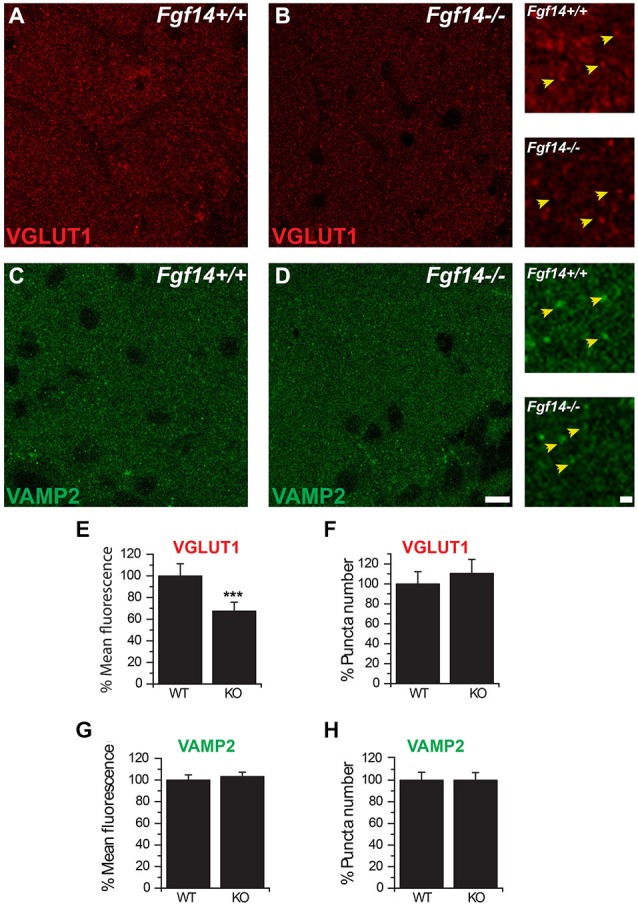
**Immunolabeling of VGLUT1 and VAMP2 at PF-Purkinje neuron synapses. (A–D)** Representative confocal images of the molecular layer in *Fgf14^+/+^* **(A,C)** and *Fgf14*^−/−^ **(B,D)** cerebellar slices showing synaptic puncta labeled with either a rabbit anti-VGLUT1, visualized with Alexa-568 conjugated secondary antibody (red), or a rabbit anti-VAMP2, visualized with Alexa-488 conjugated secondary antibody (green). The insets on the left are zoomed areas to illustrate puncta at a higher magnification. **(E,F)** VGLUT1 content per puncta is significantly decreased in *Fgf14*^−/−^ mice (*p* < 0.05, *n* = 21 images per group), while the number of puncta is unchanged (*p* > 0.05) compared to the *Fgf14*^+/+^ group. **(G,H)** VAMP2 content or puncta number are unchanged in *Fgf14*^−/−^ mice (*p* = 0.59 and *p* = 0.91, *n* = 21 images per group) compared to *Fgf14*^+/+^. Scale bar = 10 μm and 5 μm for the zoom.

Changes in the expression levels of presynaptic proteins might associate with deficits in presynaptic function. Of the proteins involved in the maintenance of the presynaptic machinery, we chose to examine the expression and pattern distribution of VGLUT1 and VAMP2 using confocal microscopy. Immunofluorescence analysis was restricted to the molecular layer, where all PF-PC synapses are located. As illustrated in Figure 3, the mean fluorescence intensity of VGLUT1 positive puncta was significantly decreased in *Fgf14^−/−^* compared to wild type mice (100.0 ± 4.7% in *Fgf14*^+/+^ vs. 65.5 ± 8.1% in *Fgf14*^−/−^ mice; *n* = 21 total images, *p* < 0.05, Student’s *t*-test), while the puncta number was unchanged (100.0 ± 12.2% in *Fgf14*^+/+^ vs. 110.0 ± 14.0 in *Fgf14*^−/−^ mice, *n* = 21 total images, *p* > 0.05, Student’s *t*-test). In contrast, neither VAMP2 puncta mean intensity nor number were changed in *Fgf14*^−/−^ compared to wild type mice (puncta mean intensity: 100.0 ± 4.7% in *Fgf14*^+/+^ vs. 103.3 ± 4.0 in *Fgf14*^−/−^ mice, *n* = 21 total images, *p* > 0.05, Student’s *t*-test; puncta number: 100.0 ± 7.3 in *Fgf14*^+/+^ vs. 100.0 ± 6.9 in *Fgf14*^−/−^ mice, *n* = 21 total images, *p* > 0.05, Student’s *t*-test).

**Figure 4:**
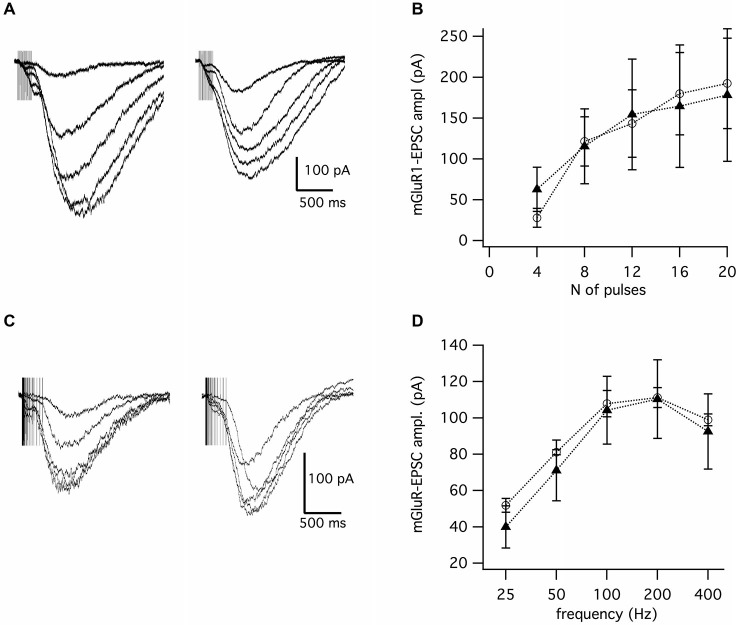
**Slow EPSCs mediated the mGluR1 receptor are not affected by genetic deletion of *Fgf14*. (A)** mGluR1-EPSCs evoked by trains of parallel fiber stimulation at 100 Hz with 4–20 pulses; left: *Fgf14*^+/+^; right: *Fgf14*^−/−^. **(B)** mGluR1-EPSC amplitude as a function of the number of pulses at 100 Hz in *Fgf14*^+/+^ (*n* = 11, open circles) and *Fgf14*^−/−^ mice (*n* = 17, filled triangles). **(C)** mGluR1-EPSCs evoked by 8 pulses of parallel fiber stimulation at frequencies from 25 to 400 Hz in *Fgf14*^+/+^ (left) and *Fgf14*^−/−^ mice (right). **(D)** mGluR1-EPSC amplitude as a function of stimulation frequency with 8 pulses in *Fgf14*^+/+^ (*n* = 9, open circles) and *Fgf14*^−/−^ mice (*n* = 15, filled triangles); data are mean ± SEM.

We then posited that these presynaptic deficits could result in reduced glutamate spill-over with consequences for activation of mGluR1, which is located peri-synaptically at these terminals and requires high-frequency release to be activated. To test this hypothesis, we applied a range of high-frequency trains from 25 to 400 Hz to evoke slow EPSCs induced by activation of mGluR1 (Tempia et al., 1998). We found that the mGluR1 responses in *Fgf14^−/−^* mice were indistinguishable from those recorded in wild type mice regardless of the number of pulses delivered (Figures 4A,B; *Fgf14*^+/+^, *n* = 11; *Fgf14*^−/−^, *n* = 17, *p* > 0.05, Student’s *t*-test) or the stimulation frequency (Figures 4C,D; *Fgf14*^+/+^, *n* = 9; *Fgf14*^−/−^, *n* = 15 each, *p* > 0.05, Student’s *t*-test).

**Figure 5:**
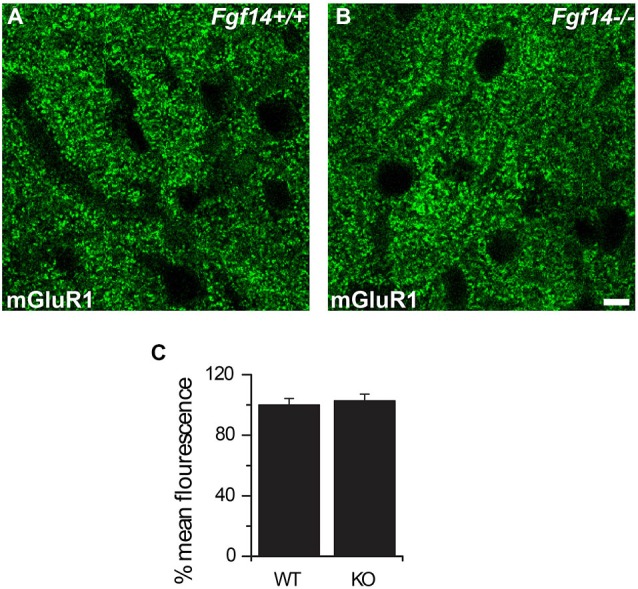
**mGluR1 expression in the molecular layer is not affected upon genetic deletion of *Fgf14***. Representative confocal images of mGluR1 expression in the molecular layer in *Fgf14*^+/+^ **(A)** and *Fgf14*^−/−^ **(B)** visualized with a rabbit anti-mGluR1 primary antibody and a secondary Alexa-488 conjugated antibody (green). **(C)** The mean fluorescence intensity value of mGluR1 in *Fgf14*^−/−^ mice (*p* < 0.05, *n* = 21 images per group) was unchanged (*p* > 0.05, *n* = 50 images per group) compared to the wild type. Scale bar = 11 μm.

The lack of changes in mGluR1 responses suggests that either the magnitude of presynaptic deficits is not sufficient to alter post-synaptic mGluR1 function or that the expression of mGluR1 is up-regulated to compensate for the presynaptic functional deficit. To test this hypothesis, we used confocal microscopy to examine the expression level and pattern distribution of mGluR1 in *Fgf14^−/−^* and wild type littermates, but we did not detect any changes across genotypes (Figure 5, *n* = 50 total analyzed images; *p* > 0.05, Student’s *t*-test).

**Figure 6:**
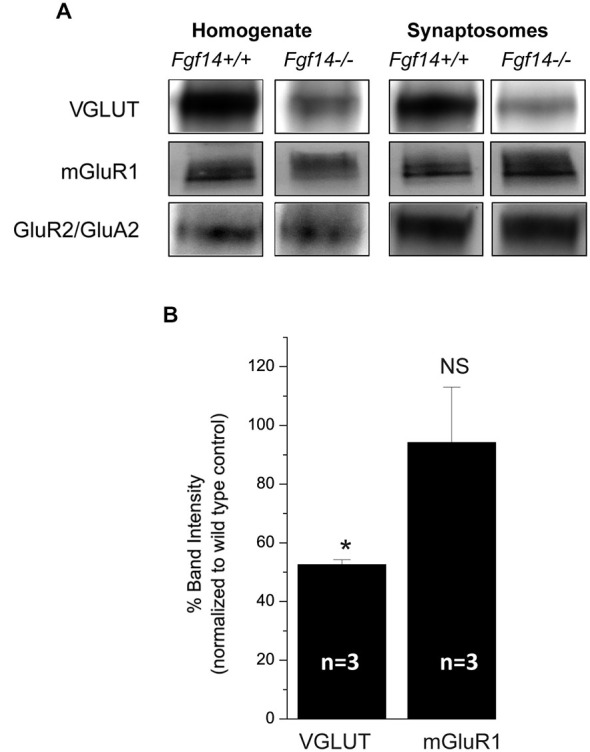
**Synaptic proteins expression in cerebellar synaptosomes. (A)** Immunoblot detection of the indicated synaptic proteins (presynaptic VGLUT1 and postsynaptic mGluR1 and GluA2) in whole cerebellum homogenates and in synaptosomes from *Fgf14^−/−^* mice and *Fgf14*^+/+^ controls. **(B)** Quantification of expression levels of VGLUT1 and mGluR1 in synaptosomes in three pairs of *Fgf14*^−/−^ mice and *Fgf14*^+/+^ controls. Mean band intensity for *Fgf14*^−/−^ extracts is plotted as a percentage of mean *Fgf14*^+/+^ band intensity (mean ± SEM). Note the significant reduction in the level of VGLUT1 (53 ± 2% of control, *p = 0.02), but not of mGluR1 (94% ± 18% of control, *p = 0.83).

To provide additional validation of our studies, we analyzed the expression of VGLUT1 and mGluR1 in synaptosomal membrane preparations from cerebellar tissue isolated from three pairs of *Fgf14*^+/+^ and *Fgf14*^−/−^ mice. As shown in Figure 6, VGLUT1 was significantly decreased at *Fgf14*^−/−^ synapses compared to *Fgf14*^+/+^ controls (*p* < 0.05), while neither mGluR1 nor GluA2, a subunit of the post-synaptic glutamate receptors, were affected by genetic deletion of *Fgf14*.

Overall, these results corroborate the hypothesis that genetic deletion of *Fgf14* results in presynaptic deficits that can explain the reduction in fast synaptic transmission responses and the increase in short-term plasticity as a base of motor deficits in this SCA27 murine model.

## Discussion

Our results demonstrate that genetic deletion of *Fgf14* leads to impairment of synaptic transmission at the PF to Purkinje neuron synapse, phenotypes that might contribute to the cerebellar ataxia observed in the SCA27 human disease. At these inputs, we found that the AMPA-R mediated EPSCs in *Fgf14*^−/−^ mice are reduced in size at most stimulation strengths, suggesting that these synapses underperform over a wide range of synaptic stimuli in the knock-out animals. To gain further knowledge of the mechanism of action underlying this phenotype, we applied paired stimulations at variable inter-pulse intervals. Consistent with a presynaptic deficit, we observed a potentiation of PPF, more evident at low inter-pulse intervals.

To confirm a presynaptic deficit at the PF-PC synapse, we analyzed the expression level and pattern distribution of the presynaptic marker VGLUT1, the vesicular glutamate transporter selectively expressed at parallel fiber synaptic varicosities (Hioki et al., 2003). We found that the number of VGLUT1 positive puncta, each representing a synaptic varicosity, was conserved in *Fgf14^−/−^* mice, but the average intensity of labeling was weaker. This phenotype indicates that in *Fgf14*^−/−^ mice the number of PF presynaptic boutons is intact, but loaded with less VGLUT1. Such outcome might result from fewer VGLUT1 molecules/per vesicle or from reduced number of vesicles per bouton. To distinguish between these two alternatives, we performed quantal analysis of Sr^2+^-induced asynchronous PF-EPSCs. This approach allowed us to selectively measure quantal events at the PF-PC synapse with minimal contamination from other synaptic inputs (i.e., the climbing fiber-PC synapse). We found that the postsynaptic response to a single quantum of glutamate released by PFs had the same size in *Fgf14*^−/−^ and *Fgf14*^+/+^ mice implying that the amount of glutamate released by a single vesicle or the number of VGLUT1 molecules per vesicle were unchanged across genotypes. Instead, this experimental outcome favors a reduced ready-releasable pool (i.e., the pool of synaptic vesicles undergoing membrane fusion in response to a single action potential) as the mechanism accounting for the synaptic deficit.

The combined increase in PPF, a form of short-term plasticity attributed to changes in residual pre-synaptic Ca^2+^ (Wu and Saggau, 1994; Debanne et al., 1996; Scullin et al., 2012; Bornschein et al., 2013), and decrease in VGLUT1 expression levels raise the question of whether these two phenotypes are functionally correlated. Recent *in vitro* studies conducted in cultured granule neurons report a role of FGF14 in regulating presynaptic voltage-gated Ca^2+^ (Cav) channels with implications for synaptic transmission (Yan et al., 2013). Similarly to our findings, these studies demonstrated suppression of EPSCs and increased PPF at granule cells to Purkinje neuron synapses upon loss of FGF14 function (Yan et al., 2013). Thus, deletion of* Fgf14* in native synapses in the knock-out mouse model, might lead to a homeostatic imbalance of presynaptic Ca^2+^ which then could trigger down regulation of VGLUT1 and potentiation of PPF.

Given the recent discovery of a role of metabotropic glutamate receptors in hereditary ataxias (Notartomaso et al., 2013), we have examined whether deficits in presynaptic release could impact postsynaptic mGluR1 activation in PC of *Fgf14^−/−^* mice. This perisynaptic mGluR requires glutamate spill-over which occurs during high-frequency train stimulation (Tempia et al., 1998, 2001). We found no changes in mGluR1 mediated responses in *Fgf14*^−/−^ mice and these observations are in agreement with our confocal studies confirming that the expression level of the mGluR1 receptor was unchanged. The preserved synaptic response mediated by perisynaptic mGluR1 receptors might appear to contradict impaired presynaptic function and reduced glutamate release. An explanation could be that the same mechanism underlying enhanced PPF might provide compensation to maintain the postsynaptic site and its perisynaptic receptors intact. In support of this, we observed that small size EPSCs evoked by a given stimulus were typically followed by much larger amplitude evoked events in *Fgf14*^−/−^ compared to wild type mice (Figure 1C). This might imply that upon repetitive stimulation *Fgf14*^−/−^ synapses could maintain synaptic ambient glutamate at a level sufficiently high to ensure full activation of perisynaptic mGluR1.

The intact mGluR1-EPSC response is indicative of normal presynaptic action potential propagation, despite the reported deficits in intrinsic excitability in *Fgf14^−/−^* granule cells, suggesting functional segregation of the FGF14 protein to distinct subcellular compartments such as the AIS, impacting initiation of the action potential (Goldfarb et al., 2007) and the presynaptic terminal, with effects on neurotransmitter release. Whether these two functions of FGF14 are part of a homeostatic regulatory loop linking intrinsic excitability and neurotransmitter release would need to be determined.

Overall, our findings further corroborate the multifaceted role of FGF14 as a global presynaptic organizer (Xiao et al., 2007; Yan et al., 2013), a Nav channel regulator (Lou et al., 2005; Laezza et al., 2007, 2009; Dover et al., 2010; Goldfarb, 2012; Shavkunov et al., 2012, 2013; Xiao et al., 2013; Bosch et al., 2015), and a kinase signaling scaffold (Shavkunov et al., 2012, 2013; Hsu et al., 2015), extending the repertoire of functions of iFGF in the normal and diseased brain (Itoh and Ornitz, 2008; Hsu et al., 2014; Pablo and Pitt, 2014).

## Author Contributions

All authors contributed to the design of the work, the acquisition, analysis, interpretation of the data and the drafting of the manuscript. FT and EH conducted and analyzed electrophysiological experiments, GN, MAA, and TKA acquired and analyzed confocal images; MAA and TKA supervised and maintained the animal colony and the animal genotyping in the USA, while EH maintained the corresponding colony in Italy. FL and FT designed the work, supervised data analysis, acquisition and interpretation and wrote the manuscript.

## Conflict of Interest Statement

The authors declare that the research was conducted in the absence of any commercial or financial relationships that could be construed as a potential conflict of interest.
